# Laparoscopic complete excision of an enormous simple hepatic cyst occupying the entire abdomen in a child: a case report and literature review

**DOI:** 10.1186/s40792-022-01445-2

**Published:** 2022-05-06

**Authors:** Nozomi Matsushita, Kenitiro Kaneko, Shoko Kato, Takayuki Odashima, Remi Kondo, Takahiro Fukuyama, Takuya Saito, Yasuyuki Fukami, Shunichiro Komatsu, Tsuyoshi Sano

**Affiliations:** Division of Gastroenterological Surgery, Department of Surgery, Aichi Medical University, 1-1 Yazakokarimata, Nagakute, Aichi 480-1195 Japan

**Keywords:** Enormous abdominal cyst, Simple hepatic cysts, Laparoscopic excision, Children

## Abstract

**Background:**

Simple hepatic cysts are common lesions in adults, but rare in children. Because of their benign nature, simple hepatic cysts may not be detected until they grow too large to be diagnosed and resected in a minimally invasive manner.

**Case presentation:**

An 18-month-old girl presented with an enormous cyst occupying the entire abdomen. The beak sign on computed tomography revealed the hepatic origin of the cyst. The cyst was decompressed through the umbilicus, which was opened by the three-triangular-skin-flap technique, thus creating a working space that enabled laparoscopic surgery. The cyst was excised en bloc together with the attached hepatic parenchyma.

**Conclusions:**

Giant simple hepatic cysts occupying the entire abdomen are rare in children. Of 14 reported cases, only 1 underwent laparoscopic treatment. We have herein reported another case of a giant simple hepatic cyst in which the beak sign on imaging and the three-triangular-skin-flap umbilical opening technique were useful for its diagnosis and laparoscopic excision, respectively. Complete excision is desirable because there is a possibility of recurrence or other diseases that require total removal, including hydatid cysts and mesenchymal hamartomas.

## Background

A simple hepatic cyst, also called a solitary nonparasitic cyst of the liver, is a congenital cyst with a fibrous wall lined by a simple cuboidal, columnar, or rarely squamous epithelium [1–4]. Such cysts are usually unilocular and are presumed to arise from isolated aberrant bile ducts [5]. Simple hepatic cysts are common lesions in adults, especially adults over 40 years of age, with an incidence ranging from 2.5 to 18.0% [1, 2, 6]. However, very few affected adults develop symptoms that require treatment. Simple hepatic cysts are rarely found in children. In the recent years, they have become increasingly detected antenatally because of the widespread use of maternal ultrasonography [6]. Because of their benign nature, simple hepatic cysts are not detected until they grow too large to be easily diagnosed and treated with minimally invasive procedures. We encountered such a case involving an 18-month-old child with an enormous cyst occupying the entire abdomen; however, the lesion was preoperatively diagnosed and completely resected by laparoscopic surgery.

## Case presentation

**Fig. 1:**
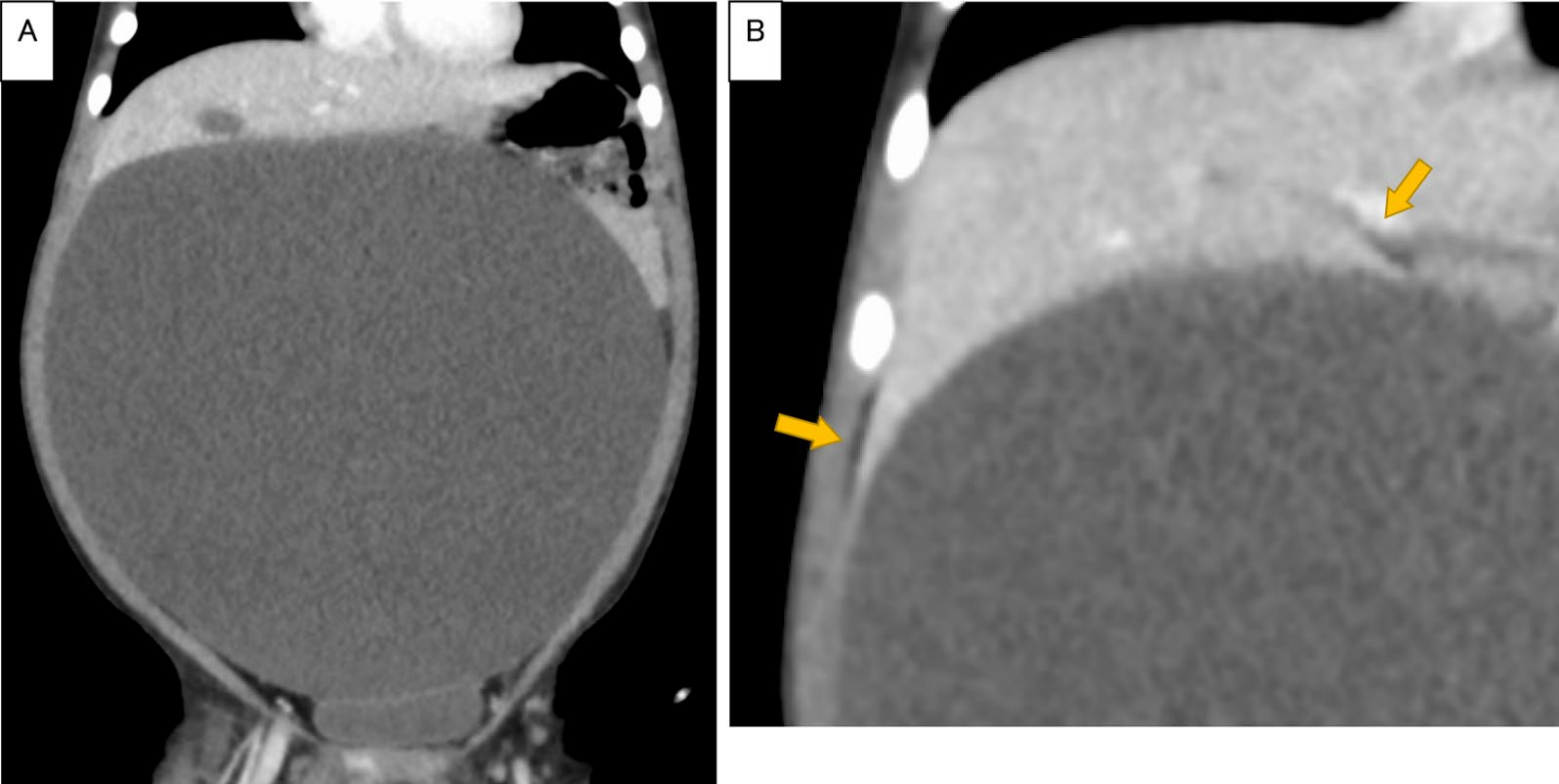
Dynamic computed tomography image of a huge abdominal cyst. The wall was thin, smooth, and not enhanced by contrast agent. The beak sign was evident, indicating a hepatic origin (arrows)

**Fig. 2:**
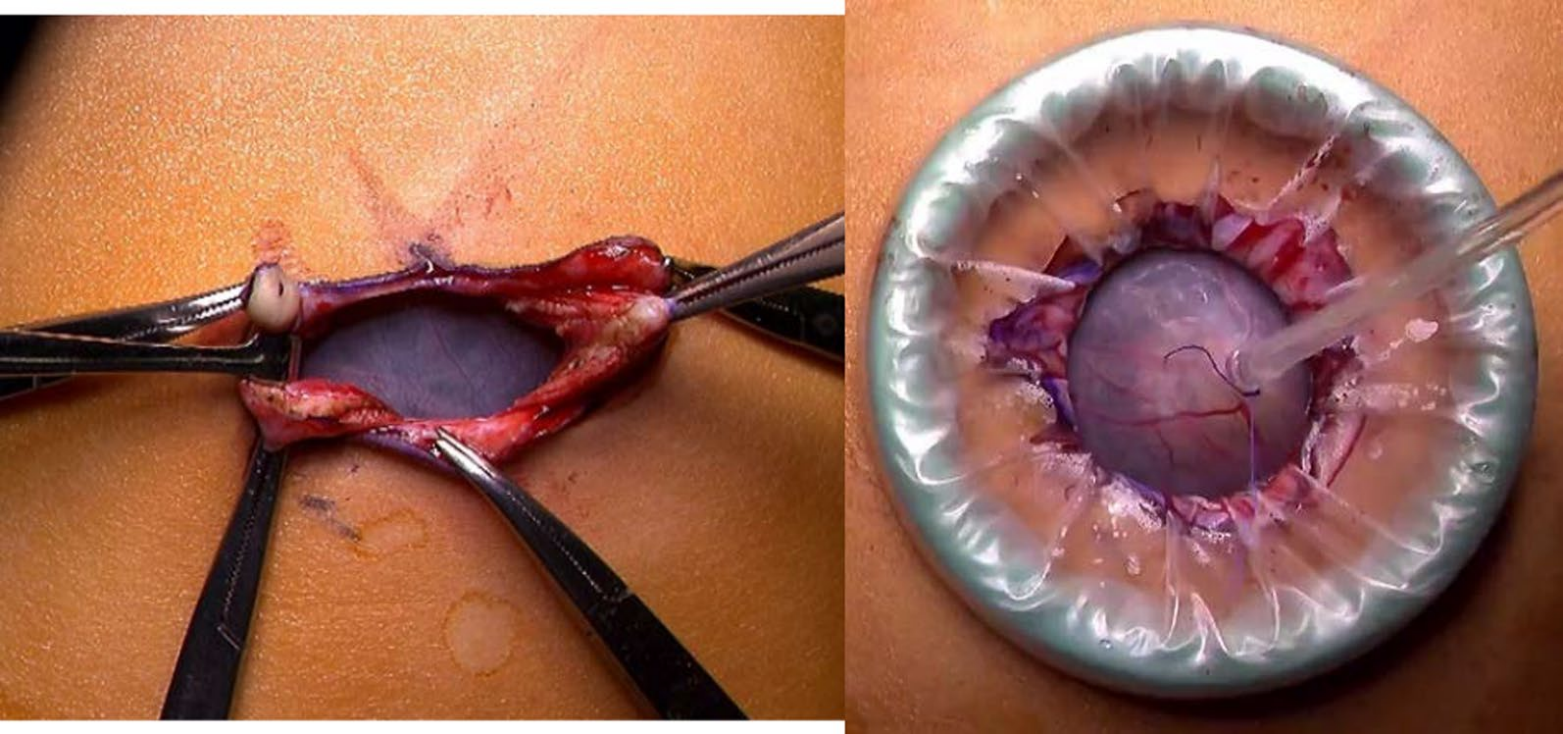
Umbilical opening technique and aspiration. The umbilicus was opened widely using the three-triangular-skin-flap technique, which created adequate exposure of the cyst wall for aspiration without spillage

**Fig. 3:**
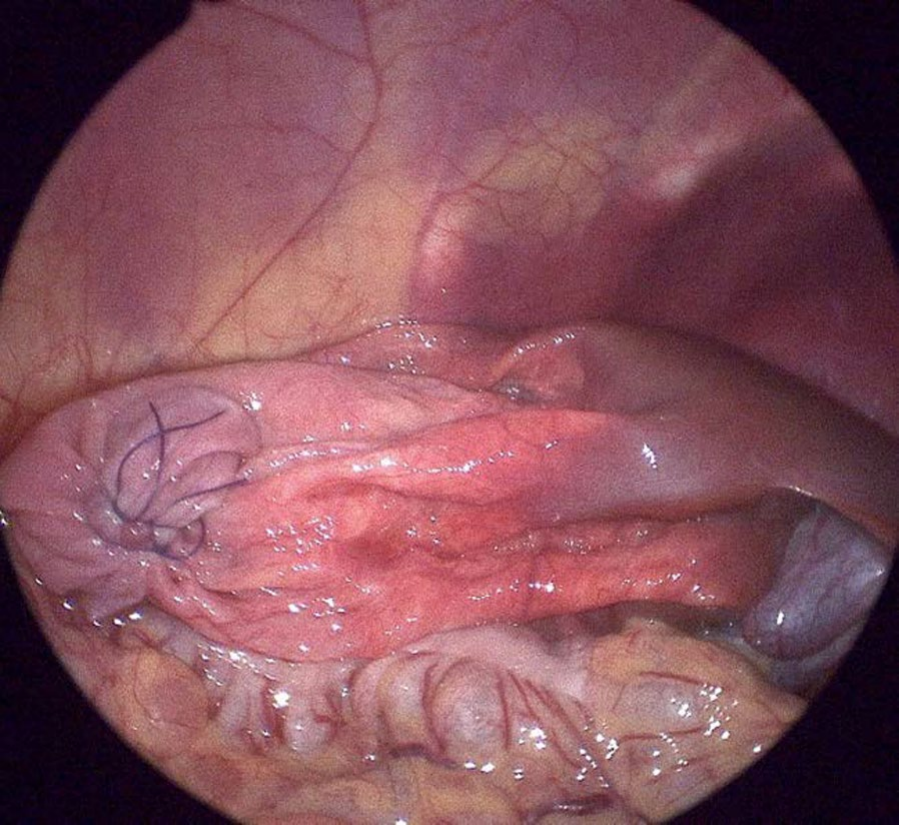
Operative findings. After decompression, laparoscopy revealed that the cyst originated from the inferior surface of liver segments 5 and 6

**Fig. 4:**
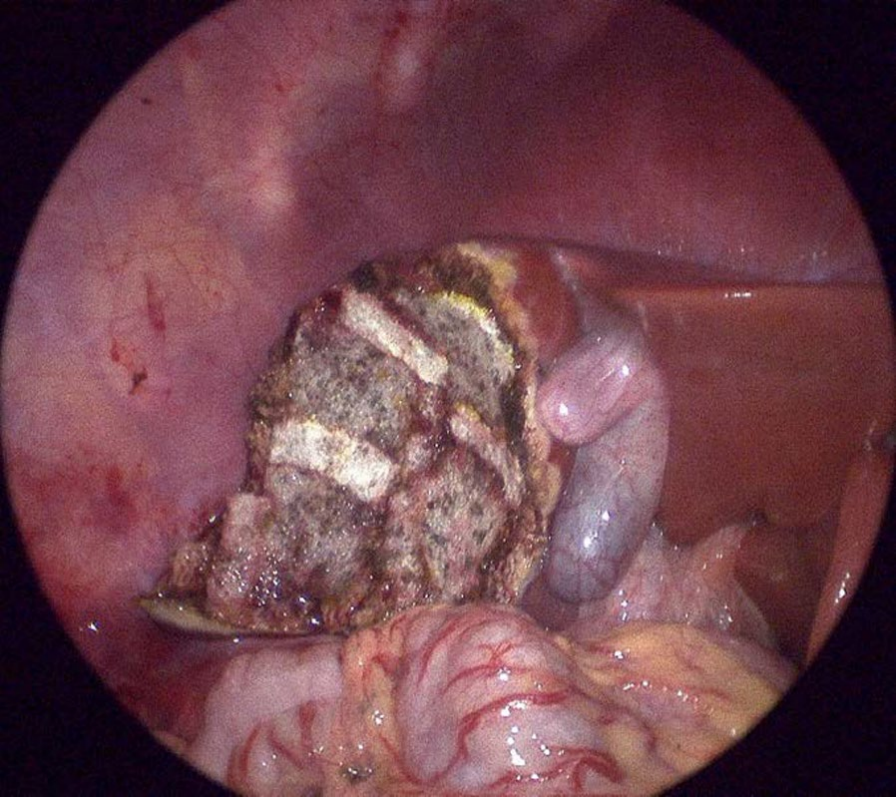
Post-excision view. The cyst with attached hepatic parenchyma was completely removed. The resected surface was covered with a tissue-sealing sheet (TachoSil; CSL Behring KK, Tokyo, Japan)

**Fig. 5:**
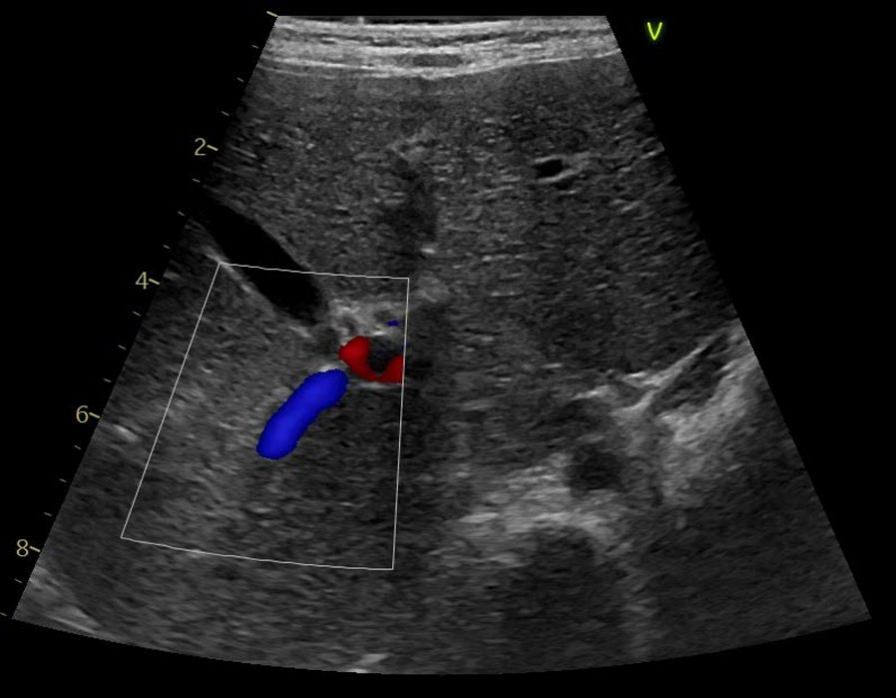
Ultrasonography 1 year after the operation showed a good portal flow at the posterior branch and no liver atrophy

**Fig. 6:**
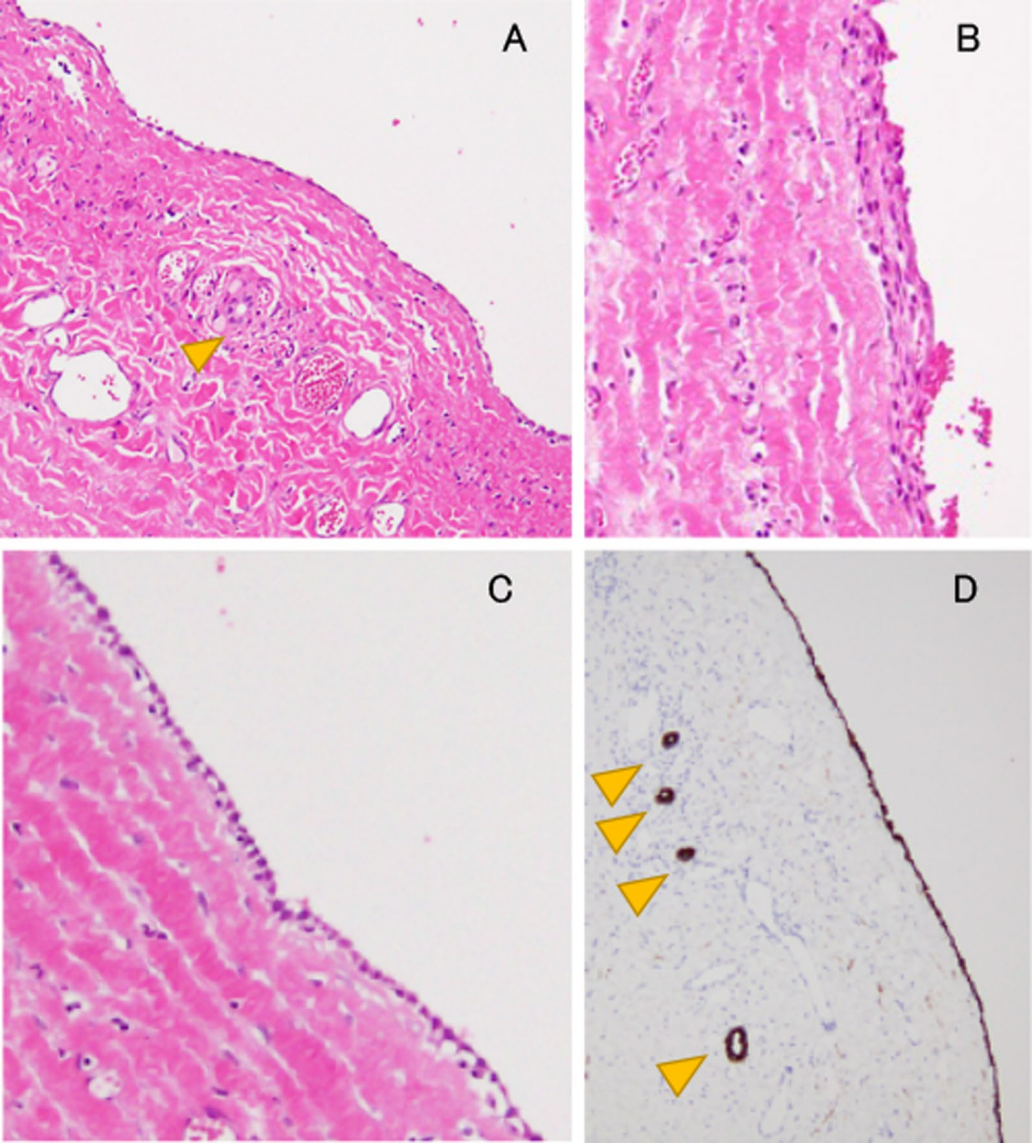
Pathological findings. **A** Most of the cyst wall was lined by a simple flattened epithelium; a few parts were lined by a **B** stratified squamous epithelium and **C** cuboidal epithelium. **D** Small bile duct-like structures (arrowheads) were positive for cytokeratin 7, as were the cyst epithelia, suggesting an aberrant bile duct origin of the cyst and squamous metaplasia

An 18-month-old girl presented with abdominal distension without abdominal pain. A cystic mass was palpable over the whole abdomen without tenderness. There were no other symptoms caused by the mass effect. Ultrasonography revealed a large unilocular, sonolucent cyst. Abdominal computed tomography (CT) showed that an enormous unilocular cyst occupied the entire abdomen (Fig. 1A). CT also demonstrated the beak sign, revealing the hepatic origin of the cyst, and the diagnosis of a simple hepatic cyst was made (Fig. 1B). The cyst was located at the periphery of segments 5 and 6. Cyst excision was planned with a minimally invasive technique. The umbilicus was opened using the three-triangular-skin-flap approach [7]. A purse–string suture was placed on the partially exposed cyst, and a catheter was inserted without spillage (Fig. 2). In total, 1520 mL of yellow serous fluid was aspirated. The cystic fluid did not contain bile, with the total bilirubin level of 0.16 mg/dL and the direct bilirubin level of 0.04 mg/dL. This decompression created a large working space that enabled laparoscopic surgery. A single-port laparoscopic surgery device was applied to the umbilicus, and another 3-mm port was placed in the right lower abdomen. The cyst originated from segments 5 and 6 (Fig. 3). Using an ultrasonic coagulation incision device (Sonicision; Medtronic, Minneapolis, MN, USA), the cyst was excised en bloc together with the attached hepatic parenchyma (Fig. 4). The operating time was 125 min, and the blood loss was 50 g. The patient was discharged on the 4th postoperative day with no complications. She was well at the 1-year follow-up. Doppler ultrasonography showed no disturbance of hepatic flow (Fig. 5). Pathologic examination showed that most of the cyst wall was lined by a simple flattened epithelium. Immunohistochemical staining showed that the cyst epithelia were positive for cytokeratin 7, but negative for estrogen receptor (Fig. 6).

## Discussion

We searched PubMed using the terms “simple hepatic cyst,” “simple liver cyst,” “nonbile containing intrahepatic cyst,” or “solitary nonparasitic cyst” and “pediatric” or “children.” We also searched Ichushi-Web using corresponding Japanese terms. The references of each article were searched for the complete collection. The criterion for enormousness was that the cyst extended into the pelvis and the transverse diameter was more than 75% of the abdominal cavity. The cases collected through this search are summarized in Table 1.

**Table 1:** Cases of enormous simple hepatic cysts in children

Case no	Year	Author	Age at detection	Age at operation	Sex	Symptoms	Preoperative diagnosis	Maximum size/volume	Location	Intervention	Outcome (follow-up)
1	2012	Oh et al. [4]	22 w gestation	8 d	F	AD	Hepatic cyst	10 cm/ND	Right lobe	Laparoscopic deroofing	Well (6 mo)
2	2020	Allan et al. [6]	24 w gestation	19 d	F	AD	Hepatic cyst	> 10 cm/500 mL	Umbilical fissure	Complete Ex	Well (2 y)
3	2020	Allan et al. [6]	30 w gestation	2 d	F	AD, respiratory distress	Hepatic cyst	12 cm/800 mL	Segment 2	Antenatal aspiration, deroofing	Well (3 y)
4	2012	Sauvat et al. [8]	33 w gestation	7 d	M	AD	Hepatic cyst	7.5 cm/ND	Right lobe	Deroofing	Well (ND)
5	1986	Michel et al. [9]	39 w gestation	0 d	F	AD, respiratory distress	Hepatic cyst	13 cm/ND	Left lobe	Cesarian section due to AD Complete Ex	Well (ND)
6	2000	Shankar et al. [10]	Antenatal	1 d	F	AD, vomiting, respiratory distress	Abdominal cyst	20 cm/ND	Right lobe	Deroofing	Well (ND)
7^a^	1990	Merine et al. [11]	0 d	0 d	F	AD, respiratory distress	Hepatic cyst	14 cm/400 mL	Right lobe	Complete Ex	Well (1 y)
8	2016	Bhosale and Singh [12]	0 d	3 d	M	AD, respiratory distress	Enteric duplication	15 cm/600 mL	Umbilical fissure	Deroofing	Operational death
9	1991	Kouchi et al. [13]	30 d	35 d	F	AD, feeding intolerance	ND	17 cm/250 mL	Both lobes	Deroofing	Well (3 y)
10^b^	1974	Saboo et al. [14]	3 mo	3 mo	F	AD, feeding intolerance, respiratory distress	Hydronephrosis or lymphatic cyst	ND/1.7 L	Left lobe	Complete Ex	Well (ND)
11	1982	Hashimoto et al. [15]	5 mo	5 mo	F	AD	Hepatic cyst	14 cm/ND	Both lobes	Deroofing	ND
12	2021	Present case	18 mo	18 mo	F	AD	Hepatic cyst	17 cm/1.5 L	Segment 5, 6	Laparoscopic complete Ex	Well (1 y)
13	1995	Pul and Pul [3]	18 mo	22 mo	F	AD	Hepatic cyst	20 cm/ND	Both lobes	Deroofing	Well (7 y)
14	2013	Banerjee and Lakhoo [16]	4 y	4 y	F	AD, abdominal pain, vomiting	Mesenteric cyst	19 cm/2 L	Right lobe	Deroofing	Well (1 y)
15	2001	Charles et al. [17]	8 y	8 y	F	AD, abdominal pain	Ovarian cyst	30 cm/ND	Right lobe	Complete Ex	ND

*w* weeks, *d* days, *mo* months, *y* years, *F* female, *M* male, *AD* abdominal distension, *ND* not described, *Ex* excision

^a^Possible mesenchymal hamartoma

^b^Multilocular cyst

Reports describing 14 children aged ≤ 15 years with simple hepatic cysts occupying the entire abdomen were collected (Table 1) [3, 4, 6, 8–17]. In total, 15 cases (including ours) were analyzed. Our case involved one of the three largest cysts. In addition to abdominal distention, six children presented respiratory symptoms due to compression. There was predominance of female sex (13:2) and perinatal cases (9:6). Preoperative diagnosis was possible in nine cases, but was difficult especially in postnatal cases, in which the cysts were already huge at the time of their discovery. These enormous cysts press other organs and obscure their organ of origin. Among various types of giant abdominal unilocular cysts, ovarian cysts, enteric duplication cysts, omental cysts, hydronephrosis, and choledochal cysts are more common in children [10, 17, 18]. In our case, the beak sign on the CT image proved the hepatic origin of the cyst (Fig. 1). Unilocular large hepatic cysts in children may be simple hepatic cysts, hydatid cysts [19], or, in exceptional cases, mesenchymal hamartomas [20–22]. Unlike simple cysts, hydatid cysts and mesenchymal hamartomas must be completely excised because spillage of hydatid fluid may cause serious anaphylactic reactions or secondary echinococcosis [19]; additionally, the residual mesenchymal hamartoma has a risk of malignant transformation into undifferentiated embryonal sarcoma [16]. Hydatid cysts are usually diagnosed by serology [1, 16]. However, in cases of negative serologic results, large unilocular hydatid cysts may reportedly be mistaken for simple hepatic cysts [19]. A mesenchymal hamartoma is usually multicystic and diagnosed by thick septa and solid areas on imaging [16, 22]. However, reports have described an enormous unilocular cystic variant of mesenchymal hamartoma that cannot be distinguished from a huge simple hepatic cyst [20–22]. In one case in the present literature review, the cyst wall contained mesenchymal tissue, and the lesion might have been a mesenchymal hamartoma [11] (Table 1).

Percutaneous aspiration of the cyst results in universal recurrence, but aspiration may be appropriate as a temporary procedure for fetuses or neonates with life-threatening symptoms [1, 4, 6]. Laparoscopic deroofing has been a preferred treatment of simple hepatic cysts [1, 2, 16, 19]. However, deroofing reportedly has a symptomatic recurrence rate of 9.6% [2]. To avoid recurrence, some surgeons apply omentopexy or methods that destruct the epithelial lining, including ethanol sclerotherapy, electrocautery coagulation, and argon beam coagulation; however, the effectiveness of these techniques lacks evidence [2]. Leaving a part of the cyst, especially a part lined with a squamous epithelium, cannot eliminate the risk of malignant transformation [3, 23]. The squamous epithelium with additional stratified changes in our case seemed to be metaplasia from biliary epithelia due to intracystic pressure (Fig. 5). Preoperative examinations cannot exclude the possibility of a hydatid cyst or mesenchymal hamartoma, which requires complete removal [19–22]. Complete excision is desirable when feasible. A minimally invasive approach is difficult for children with giant cysts because of the limited working space. Among the collected cases in this literature review, only one other case besides ours adopted a laparoscopic approach. Our technique for opening the umbilicus provided a large enough field to place a purse–string suture for aspiration without spillage (Fig. 2) [7]. This reduction allowed a large working space, which facilitated laparoscopic complete excision.

## Conclusions

Enormous simple hepatic cysts in children are rare and difficult to diagnose and treat by minimally invasive techniques. Only 14 cases have been reported in the literature, showing female and perinatal predominance. The present report is the 15th case and involved an 18-month-old girl in whom the beak sign on imaging designated a hepatic origin of the cyst. The three-triangular-skin-flap umbilical opening technique enabled aspiration without spillage and laparoscopic complete excision. Complete excision is desirable when feasible because there is a possibility of recurrence or other diseases that require total removal.

## Data Availability

Data sharing is not applicable to this article because no datasets were generated or analyzed during the current study.

## Abbreviation

CT: Computed tomography
